# Comparison of the effects of symmetric and asymmetric temperature elevation and CO_2_ enrichment on yield and evapotranspiration of winter wheat (*Triticum aestivum* L.)

**DOI:** 10.1002/ece3.1081

**Published:** 2014-04-22

**Authors:** Yunzhou Qiao, Huiling Liu, Seppo Kellomäki, Heli Peltola, Yueyan Liu, Baodi Dong, Changhai Shi, Huizhen Zhang, Chao Zhang, Jinnan Gong, Fuyan Si, Dongxiao Li, Xin Zheng, Mengyu Liu

**Affiliations:** 1 Key Laboratory of Agricultural Water Resources & Hebei Key Laboratory of Agricultural Water-Saving, Center for Agricultural Resources Research, Institute of Genetics and Developmental Biology, Chinese Academy of Sciences286 Huaizhong Road, Shijiazhuang, 050021, China; 2 Shijiazhuang Center for Agricultural Product Quality inspectionShijiazhuang, 050021, China; 3 University of Eastern Finland, School of forest SciencesJoensuu, Yliopistokatu 7, Borealis Building, Box 111, Fin- 80101, Joensuu, Finland; 4 Library of Shijiazhuang UniversityShijiazhuang, 050035, China; 5 Key Laboratory of Geographic Information Science, Ministry of Education, East China Normal UniversityShanghai, 200241, China

**Keywords:** Aboveground biomass, grain yield, root biomass, soil water depletion, yield components

## Abstract

Under the changing climate, asymmetric warming pattern would be more likely during day and night time, instead of symmetric one. Concurrently, the growth responses and water use of plants may be different compared with those estimated based on symmetric warming. In this work, it was compared with the effects of symmetric (ETs) and asymmetric (ETa) elevation of temperature alone, and in interaction with elevated carbon dioxide concentration (EC), on the grain yield (GY) and evapotranspiration in winter wheat (*Triticum aestivum* L.) based on pot experiment in the North China Plain (NCP). The experiment was carried out in six enclosed-top chambers with following climate treatments: (1) ambient temperature and ambient CO_2_ (CON), (2) ambient temperature and elevated CO_2_ (EC), (3) elevated temperature and ambient CO_2_ (ETs; ETa), and (4) elevated temperature and elevated CO_2_ (ECETs, ECETa). In symmetric warming, temperature was increased by 3°C and in asymmetric one by 3.5°C during night and 2.5°C during daytime, respectively. As a result, GY was in ETa and ETs 15.6 (*P* < 0.05) and 10.3% (*P* < 0.05) lower than that in CON. In ECETs and ECETa treatments, GY was 14.9 (*P* < 0.05) and 9.1% (*P* < 0.05) higher than that in CON. Opposite to GY, evapotranspiration was 7.8 (*P* < 0.05) and 17.9% (*P* < 0.05) higher in ETa and ETs treatments and 7.2 (*P* < 0.05) and 2.1% (*P* > 0.05) lower in ECETs and ECETa treatments compared with CON. Thus, GY of wheat could be expected to increase under the changing climate with concurrent elevation of CO_2_ and temperature as a result of increased WUE under the elevated CO_2_. However, the gain would be lower under ETa than that estimated based on ETs due to higher evapotranspiration.

## Introduction

Atmospheric CO_2_ concentration has increased from 280 *μ*mol·mol^−1^ prior to the industrial revolution to 379 *μ*mol·mol^−1^ until 2005 and may reach 700 *μ*mol·mol^−1^ by the end of the 21st century (IPCC 2007). Such an increase in CO_2_ concentration might trigger a rise in global temperature by 1.4–5.8°C (IPCC 2001). As a result, growth of different plants will inevitably be affected because prevailing temperature and atmospheric CO_2_ concentration together with water availability affect the physiological processes of plants (i.e., photosynthesis, respiration, and transpiration, Abou-Hussein 2012).

Any negative impacts of foreseen climatic change might affect also the availability and quality of food crops such as winter wheat (*Triticum aestivum* L.), which is one of the most important food crops not only in the North China Plain (NCP) but also elsewhere around the world. Based on previous studies, the elevation of CO_2_ alone has been expected to increase significantly the grain yield and the water use efficiency in wheat (Kimball and Idso 1983; Morison 1985; Drake et al. 1997; Amthor 2001; Polley 2002; Guo et al. 2010; Qiao et al. 2010). This was related to the increase in photosynthesis and decrease in transpiration due to reduced stomatal aperture (Kimball and Idso 1983). The concurrent elevation of CO_2_ has also been suggested to at least partly compensate the negative effects of elevated temperature (Lawlor and Keys 1993; Lal et al. 1998, 1999; Challinor and Wheeler 2008).

However, the most previous studies in winter wheat have assumed equal elevation of temperature during daytime and nighttime (Peng et al. 2004; Lobell 2007; Fang et al. 2012). This was despite the meteorological observations and model-based predictions since 1990s which suggested asymmetric temperature increase (ETa), that is, the increase will be higher in nighttime than in daytime (Karl et al. 1993; IPCC 2001, 2007). For example, in China, the mean daily minimum temperature (night time) has increased 2–3 times more than the maximum temperature (day time) during the past five decades (Ren et al. 2005; Tan et al. 2009).

Growth responses to the asymmetric increase in minimum and maximum diurnal temperature have been so far studied mainly by using crop modeling, and particularly in food crops such as rice (*Oryza sativa* L., Peng et al. 2004; Lobell 2007; Mohammed and Tarpley 2009; Dong et al. 2011; Zhang et al. 2013), maize (*Zea mays* L., Dhakhwa and Campbell 1998), and soybean (*Glycine max* (L.) Merr., Dhakhwa and Campbell 1998). To date, only few studies have addressed the response of wheat to higher night warming without daytime warming (Rosenzweig and Tubiello 1996; Dhakhwa and Campbell 1998; Lobell 2007; Lobell and Ortiz-Monasterio 2007; Prasad et al. 2008; Fang et al. 2012). In general, the physiological and ecological activities of prime importance for yield and water use of crops (i.e., photosynthesis, respiration, and transpiration) occurred mainly during daytime excluding respiration in night (Xia et al. 2009). Higher elevation of temperature during nighttime might increase respiration losses of photoassimilates (Abou-Hussein 2012) and decrease crop yield compared with symmetric warming in daytime and nighttime (Lobell et al. 2011). However, it was still unclear how asymmetric and symmetric warming in combination with elevated CO_2_ might affect the yield and water use of winter wheat.

In the above context, the aim of this work was to compare the effects of symmetric and asymmetric elevation of temperature alone and in interaction with elevated CO_2_ concentration, on the grain yield and evapotranspiration in winter wheat (*Triticum aestivum* L.) in the North China Plain (NCP). It was hypothesized that the grain yield of winter wheat could increase less in the future under the concurrent elevation of CO_2_ and asymmetric elevation of temperature than that with symmetric warming.

## Materials and Methods

### Climate treatments and plant material

#### Climate treatments

The study was conducted at the Luancheng Agro-Eco Experimental Station, Chinese Academy of Sciences (37.53^°^N, 114.41^°^E; altitude 50.1 m a.s.l), in the North China Plain (NCP). The long-term means of annual temperature and precipitation during the past 30 years were 12.2°C and 530 mm, respectively. To study the effects of symmetric (ETs) and asymmetric (ETa) elevation of temperature alone and in interaction with elevated CO_2_ concentration, on the grain yield and evapotranspiration in winter wheat, Six enclosed-top chambers were randomly assigned to ambient (AC: 396.1 ± 29.2 *μ*mol·mol^−1^) or elevated (EC: 760.1 ± 36.1 *μ*mol·mol^−1^) CO_2_ concentration in combination with three temperature regimes (Ambient temperature; ETs - symmetric elevation of 3°C; ETa - asymmetric elevation of 3°C, i.e. +3.5°C during night, +2.5°C during daytime). As a result, we had following climate treatments: ambient conditions (CON), symmetrically elevated temperature (ETs), asymmetrically elevated temperature (ETa), elevated CO_2_ (EC), elevated CO_2_ and symmetrically elevated temperature (ECETs), elevated CO_2,_ and asymmetrically elevated temperature (ECETa).

For this work, winter wheat (*T. aestivum* L. cv. Jimai22) was sown at the experimental station around the chambers on 10 October 2010. Eighteen plants of uniform size and with three tillers were selected and transplanted on 20 March 2011 to 60 stainless steel pots (diameter = 28 cm, depth = 30 cm), after their roots were pruned to similar size (plants has reached the turning green stage). The 60 pots were randomly assigned to the six chambers (*n* = 10 pots per chamber). Six pots in each chamber were sampled regularly every 5 day, and the remaining four pots were left for final harvest and determination of water use. Each pot was filled with 20 kg foam soil from the experimental station and saturated with water after transplanting. The soil was sieved (four mesh) and mixed homogeneously with N fertilizers (1.5 g urea). The total N and available N, P, and K contents were 93 mg·g^−1^, 77.49 mg·kg^−1^, 47.6 mg·kg^−1^, and 35.8 mg·kg^−1^, respectively. Each pot was top-dressed with 1.5 g urea at the jointing stage. All pots were weighed and irrigated every 3 day to maintain soil water content between 60% and 70% of field water capacity.

### Chamber design and performance

#### Chamber design

**Figure 1:**
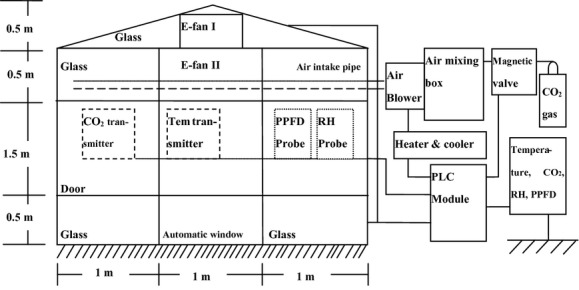
Side view and main components of the enclosed-top chamber applied in the present study. E-fan I are two electronic fans that are fixed on the east and west top walls to bring the out cooler air in when the temperature in chambers is out of controlling capacity of freezers. The automatic window will be opened and closed together with E-fan I being turned on or off for the same purpose. E-fan II is the electronic fan that is fixed on the top facing ground to mix air in the chambers. The air intake pipe, extending along the four walls (totally 12 m), is 2 m above the ground and dotted with holes every 20 cm.

The chamber systems consisted of two parts, a frame and a controlling system, containing CO_2_ and temperature controls system (Fig. 1). The frame was made of aluminum alloy with 3-mm-thick glass walls. The ground area was 9 m^2^ (3 m × 3 m) and the height was 2.5 m, resulting in interior chamber volume of 22.5 m^3^. A triangular prism top (3 m × 3 m × 0.5 m) made of 3-mm-thick glass was placed above the chamber frame. One electronic fan (E-fan I in Fig. 1) was attached to each end (east end and west sides) of the prism (facing the inside to bring cooler air into the chamber from outside). An additional fan (E-fan II in Fig. 1) was attached to the top of each chamber facing the ground surface (crops) to mix air within the chamber. A CO_2_ transmitter (BS03II; Hanwei Corporation, Zhengzhou, China), temperature transmitter (WB201; Qinming Corporation, Baoji, China), and light and humidity sensor (Hobo U1202; Onset Corporation, Cape Kod, MA) were fixed both at the center of each chamber and outside the chamber. The height of the sensors and transmitters were moved as the crops grew to observe and control the corresponding environmental parameters at the canopy.

The CO_2_ concentration inside the CO_2_-enriched chamber was continuously monitored and controlled by a CO_2_ control system, which consisted of CO_2_ gas containers, electromagnetic valves, air-mixing boxes, and CO_2_ transmitters (Fig. 1). The CO_2_ concentration inside the chamber was sensed, and the information transferred to a PLC module (XHS CS01; China), where it was compared with the target CO_2_ concentration (780 ± 20 *μ*mol·mol^−1^). When the concentration was lower than 770 *μ*mol·mol^−1^, the magnetic valve would turn on and pure CO_2_ gas would be injected into a 1-m^3^ wooden box. After being mixed with air, the CO_2_ gas was diluted to approximately 3000 *μ*mol·mol^−1^ and then injected into the CO_2_-enriched chambers by an air blower through an air intake pipe. The air intake pipe was fixed along the four sides of the glass wall 1.5 m above the ground surface and was perforated with 4-mm-diameter holes every 20 cm. With the CO_2_ concentration approached 790 *μ*mol·mol^−1^, the air blower slowed and the magnetic valve was shut off by the PLC module. The CO_2_ concentration in chambers exposed to ambient CO_2_ (AC) was not controlled.

The temperature control system (Fig. 1) consisted of a heater, compressor (freezer), temperature transmitter, and auxiliary adjusting equipment (automatic window and fixed fans). When the temperature transmitter sensed a chamber temperature that was higher or lower than the preset target temperature, the PLC module would signal to trigger the compressor or electronic heater to cool or warm the air in the chambers. The cooling and warming air entered the chamber through the same pipe as the CO_2_ gas (Fig. 1). We programmed the PLC module to open the automatic window (to let in cool air) and turn on the electronic fan (to let out warm air) when the chamber temperature was 1°C higher than the target temperature.

#### Chamber performance

**Figure 2:**
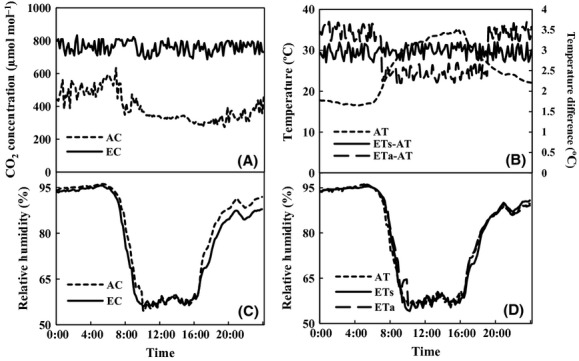
The CO_2_ concentration (A), temperature, and temperature difference (B) and the relative humidity between CO_2_ (C) and temperature (D) treatment in the enclosed-top chambers. Data shown are recorded every 10 min on 10 May 2011.

The target CO_2_ enrichment of 780 *μ*mol·mol^−1^ and temperature elevation were simulated well by the chamber systems (Fig. 2A and B). The daily average CO_2_ concentration observed was 760.1 ± 36.1 *μ*mol·mol^−1^ in CO_2_-enriched chambers (Fig. 2A). The corresponding CO_2_ concentration in AC chambers was 396.1 ± 29.2 *μ*mol·mol^−1^ (Fig. 2A). The average temperature of CON, ETs, and ETa were 25.5 ± 6.4°C, 28.5 ± 6.4°C, and 28.5 ± 6.0°C on 10 May 2011, respectively (Fig. 2B). The average temperature in ETs and ETa chamber were 2.93 ± 0.14°C and 2.95 ± 0.53°C higher than CON. The temperature of ETa is 3.45 ± 0.13°C and 2.46 ± 0.14°C higher than that of CON in nighttime and daytime, respectively (Fig. 2B). Atmospheric relative humidity (RH) and light intensity (especially the photosynthetic active radiation, PAR) were also followed, because they were closely related to leaf transpiration and photosynthetic rate. The RH of AC and EC treatment were 79.3 ± 15.8% and 77.4 ± 15.3%, respectively (Fig. 2C). The RH of AT, ETs, and ETa treatment were similar to each other (Fig. 2D). On sunny days, the total PAR inside each chamber was about 15% less than that outside the chamber. In addition, light availability was reduced in chambers compared with outside conditions especially in early morning, late afternoon, and cloudy days when light intensity was rather low.

Regarding the temperature control, the drop down of the inside temperature through exchanging with outside cooler atmosphere helped to maintain the temperature around the target temperature when the sun light is too strong in relation to the cooling and the freezing capacity (Fig. 1). The RH was also similar between the chambers with different CO_2_ concentrations and different temperatures (Fig. 2C and D). As the vapor pressure would increase with temperature elevation and the saturated water vapor pressure will increase more than the virtual vapor pressure, the RH would be decreased and the vapor pressure deficit (VPD) between leaf and air would be higher than that in the chambers with ambient temperature. The ventilation system helped to keep similar RH between chambers with different temperatures.

### Measurements

#### Grain yield and biomass

Four pots in each chamber were harvested manually at maturity (9 June 2012). All 18 plants from each pot were used to determine the mean number of spike number per unit area of ground, kernels per spike, kernel weight, and harvest index (HI). Spikes were counted before harvesting. Economic yields and total aboveground biomass were determined after air-drying to constant weight. HI was the ratio of GY to total aboveground biomass.

#### Roots characteristics

All roots in each pot were sampled to determine root biomass and length, root length density (RLD), and root/shoot ratio (RSR). After removing soil and organic debris, root length was measured according to Tennant (1975). Roots were then oven-dried at 60°C to determine dry weight. RLD was calculated as root length per liter of soil. The root/shoot ratio was calculated as the ratio of root biomass to aboveground biomass.

#### Soil moisture monitoring

Soil volumetric water contents of the pots for harvesting (*n* = 4 per treatment) was measured hourly using an FDR probe placed at mid depth in center of each pot and recorded by data logger (RHD-05; Ruihua Electronics Corporation, Handan, China) during the growing season.

### Data analyses

Evapotranspiration of the entire growing season was calculated using the water balance equation abbreviated from Allen et al. (1998):


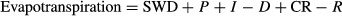
(1)

where SWD was soil water depletion (initial soil water content minus final soil water content), P was precipitation, I was irrigation, D was drainage from the root zone, CR was capillary rise to the root zone, and R was runoff. Equation (1) was simplified to equation (2) by zeroing P, D, CR, and R, because this was an enclosed-top chamber experiment with pot cultivation:


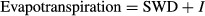
(2)

WUE was then calculated from the formula:


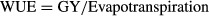
(3)

The hourly SWD was calculated as the hourly decrease of soil moisture, that is, soil moisture at *t* = *n* o'clock minus soil moisture at *t* = *n* + 1 o'clock.

Two statistical analyzing methods were applied. First, two-way ANOVA (SPSS version 11.5, SPSS, Inc., Chicago, IL) was applied to test the alone and interactive effects of CO_2_ concentration * ETs and CO_2_ concentration * ETa on grain yield, aboveground biomass, root characteristics, evapotranspiration, and water use efficiency. In this context, the effects of asymmetric and symmetric temperature elevation were considered separately. The data analyses followed a pseudo-replicated design (Ceulemans et al. 2002), in which each pot was considered as an individual replicate (thus, four replicates). Secondly, one-way ANOVA was applied to test the difference of all the investigated traits between CON, EC, ETs, ETa, ECETs, ECETa.

## Results

### Grain yield and yield components

**Figure 3:**
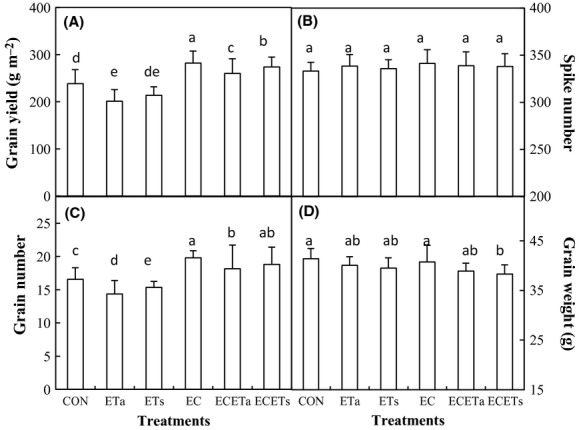
The grain yield (A), spike number (B), grain number (C), and grain weight (D) as affected by elevated CO_2_ concentration (EC, 760 *μ*mol·mol^−1^) in combination with symmetric (ETs: equally +3°C in daytime and nighttime) and asymmetric (ETa: +3.5°C during night and +2.5°C during daytime) warming. Different letters indicate statistical difference at *P* < 0.05.

Both symmetric (ETs) and asymmetric elevation (ETa) of temperature reduced GY compared with ambient conditions (CON), as a result of significant decrease in grain number (*P* < 0.05, Fig. 3A and C). Asymmetric warming reduced the GY more than the symmetric warming did. Elevation in CO_2_ alone (EC) increased GY the most compared with CON (Fig. 3A) followed by concurrent elevation of CO_2_ and symmetric warming (ECETs) and concurrent elevation of asymmetric warming (ECETa). The grain number was more sensitive to the elevation of temperature and/or CO_2_ than thousand grain weight (Fig. 3C and D). Spike number was not affected by climate treatments (*P* > 0.05, Fig. 3B), because the tiller number was already formed when chamber experiment started.

### Aboveground biomass and harvest index (HI)

**Figure 4:**
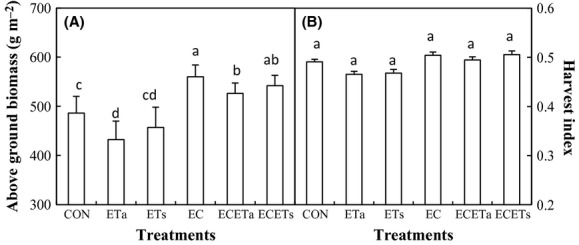
The total aboveground biomass and harvest index as affected by elevated CO_2_ concentration (EC, 760 *μ*mol·mol^−1^) in combination with symmetric (ETs: equally +3°C in daytime and nighttime) and asymmetric (ETa: +3.5°C during night and +2.5°C during daytime) warming. Different letters indicate statistical difference at *P* < 0.05.

Total aboveground biomass was also remarkably decreased under ETs and Eta compared to CON, but less than that of grain yield (Fig. 4A). Asymmetric warming decreased aboveground biomass more than symmetric warming did. Elevated CO_2_ alone increased aboveground biomass the most compared with CON, followed by ECETs and ECETa. Harvest index was not statistically affected by any of climate treatments (Fig. 4B).

### Root biomass, root length density (RLD), and root/shoot ratio (RSR)

**Figure 5:**
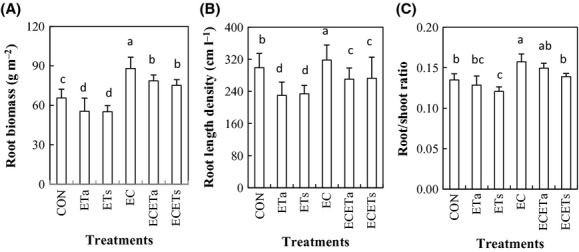
The root biomass (A), root length density (B), and root/shoot ratio (C) as affected by elevated CO_2_ concentration (EC, 760 *μ*mol·mol^−1^) in combination with symmetric (ETs: equally +3°C in daytime and nighttime) and asymmetric (ETa: +3.5°C during night and +2.5°C during daytime) warming. Different letters indicate statistical difference at *P* < 0.05.

Both asymmetric and symmetric warming reduced root biomass on average by 15.6% (*P* < 0.05) and RLD by 22.3% (*P* < 0.05) compared with CON (Fig. 5A and B). Elevation of CO_2_ alone increased the root biomass more than RLD. However, root biomass increased also in ECETa and ECETs compared with CON, opposite to RLD. Both asymmetric and symmetric elevation of temperature (alone) decreased RSR compared with CON, whereas elevation of CO_2_ (alone) increased it (Fig. 5C) as did ECETs and ECETa.

### Evapotranspiration and WUE

**Figure 6:**
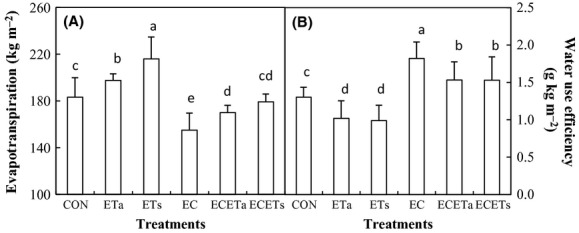
The evapotranspiration (A) and water use efficiency (B) as affected by elevated CO_2_ concentration (EC, 760 *μ*mol·mol^−1^) in combination with symmetric (ETs: equally +3°C in daytime and nighttime) and asymmetric (ETa: +3.5°C during night and +2.5°C during daytime) warming. Different letters indicate statistical difference at *P* < 0.05.

**Figure 7:**
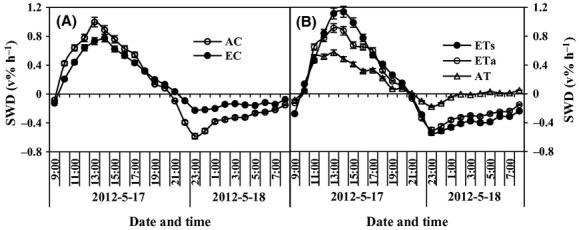
The hourly soil water depletion as affected by elevated CO_2_ concentration (EC, 760 *μ*mol·mol^−1^) (A) and symmetric (ETs: equally +3°C in daytime and nighttime) *versus* asymmetric (ETa: +3.5°C during night and +2.5°C during daytime) warming (B). AC - ambient CO_2_, AT, ambient temperature.

Both asymmetric and symmetric warming increased evapotranspiration (Fig. 6A). Water uptake was higher under ETs than under ETa. Elevation of CO_2_ (alone) decreased water consumption remarkably. Under concurrent elevation of CO_2_ and asymmetric warming, evapotranspiration decreased significantly compared with CON (*P* < 0.05), Unlike in ECETs (*P* > 0.05). The hourly soil water depletion (SWD, Fig. 7A and B) rate began to increase at 9 am, had the maxima at 1 pm and began to decrease until 8 pm. Thereafter, the SWD rate concave negative, implying that soil water was being recharged through capillary from deeper soils.

EC decreased evapotranspiration primarily during the daytime, from 10 am to 5 pm (Fig. 7A). Soil water in EC treatments was replenished overnight (from 9 pm to 8 am) through capillarity, at a lower rate than in ambient CO_2_ treatments. ETs and ETa both caused higher SWD during the daytime, but differences still existed between them (Fig. 7B). Both ETs and ETa mainly increased SWD from 12 am to 6 pm, while ETs also resulted in higher SWD than ETa from 1 pm to 4 pm. The difference in soil water loss can also be explained by capillary recharge (Fig. 7B).

WUE was reduced by ETs and ETa compared with CON, opposite not only to elevation of CO_2_ alone but also to concurrent elevation of CO_2_ and symmetric or asymmetric elevation of temperature (Fig. 6B). The two warming patterns had similar effect on WUE but due to different response degree of GY and evapotranspiration. On average, 21.8% (*P* < 0.05) decrease observed in WUE by ETa compared to CON was due to 15.6% (*P* < 0.05) decrease in GY and 7.8% (*P* < 0.05) increase in evapotranspiration. The counter part for ETs could be attributed to the 10.3% (*P* < 0.05) decrease in GY and 17.9% (*P* < 0.05) increase in evapotranspiration.

## Discussion and Conclusions

Our results indicated that asymmetric warming (alone) with slightly higher warming in nighttime compared with daytime decreased GY and total aboveground biomass more than the symmetric warming did. This was also observed by Lobell et al. (2011). This result might be explained by the increase in respiration loss of photoassimilates due to higher night warming (Abou-Hussein 2012). On the contrary to GY, in our work, evapotranspiration increased under elevated temperature. In general, under warmer climate, larger share of water from the precipitation and irrigation would be transpired into vapor before entering soil (Hou et al. 2012). Furthermore, the transpiration would increase to cool leaves (Abou-Hussein 2012). In this work, evapotranspiration increased more under asymmetric warming conditions than under symmetric warming, compared with CON. In some previous studies, no evaporative loss has been expected to occur during night (Peterson et al. 1995; Todisco and Vergni 2008 Lovelli et al. 2010). Thus, in principle, higher increase in daytime temperature could be expected to increase water consumption.

In general, a small increase in temperature could stimulate substantially crop yield when the prevailing temperature is below the optima for photosynthesis. The converse is true when temperature was near or higher than the optima (Baker and Allen 1993; Polley 2002; Ortiz et al. 2008; Abou-Hussein 2012). The concurrent elevation of CO_2_ with asymmetric or symmetric warming can partly compensate the negative effect of elevated temperature on crop yield and water use (Ludwig and Asseng 2006; Krishnan et al. 2007). In this work, both in ECETs and in ECETa, yield of wheat increased and water consumption (and evapotranspiration) decreased, compared with CON, or ETs and ETa alone.

The wheat yield is dependent on ear number (per unit ground area), grain number per ear, and individual grain mass (usually described by 1000-grain weight). According to Amthor (2001), ear number is the yield component which was consistently promoted by elevated CO_2_. Our results of grain number and thousand grain weight were well in line with findings reported by Amthor (2001). Ear number was usually developed before wintering stage. When initiated before the jointing stage, CO_2_ fumigation has shown clear effects on spike number (see many examples in Amthor 2001). In the present experiment, tiller number was not affected by elevated CO_2_ and warming patterns, which might be attributed to starting treatments after the plants turned green.

The continuous hourly detection and recording of soil moisture provided an opportunity to examine hourly water use of wheat crops, SWD and soil moisture dynamics. The elevated CO_2_ reduced SWD from 10 am to 5 pm (Fig. 7A and B). In fact, most of the plant physiological activities were affected by CO_2_ concentration only during this time, because light intensity is the driving energetic resource for leaf carbon assimilation. Also Garcia et al. (1998) reported that elevation of CO_2_ concentration to 550 *μ*mol·mol^−1^ resulted in a 28% higher midday photosynthetic rate in spring-wheat leaves, with a simultaneous 36% reduction in stomatal conductance.

In our work, evaporation and transpiration occurred also during the night, but the measured values were small in relation to increases in soil moisture due to capillarity. The negative SWD at nighttime could only be interpreted as water recharge. Even so, the hourly SWD demonstrated decrease in evapotranspiration due to CO_2_ elevation. However, in the future, the estimates of evapotranspiration should be more precise, through weighing pots hourly and the fresh weight of plant material being precisely subtracted from each measurement.

To conclude, both asymmetric and symmetric warming could reduce wheat yield and enhance evapotranspiration compared with CON as was demonstrated in our work. The asymmetric warming could also result in larger reduction in GY than that based on symmetric warming. However, the crop yield of wheat could be expected to increase under the changing climate with concurrent elevation of CO_2_ and temperature as a result of increased WUE under the elevated CO_2_. But the expected increase would be surely lower under ETa than that estimated based on ETs due to higher evapotranspiration.
